# Medical Assistance in Dying: A Review of Canadian Health Authority Policy Documents

**DOI:** 10.1177/23333936231167309

**Published:** 2023-05-06

**Authors:** Robyn Thomas, Barbara Pesut, Gloria Puurveen, Sally Thorne, Carol Tishelman, Betsy Leimbigler

**Affiliations:** 1 University of British Columbia Okanagan, Kelowna, Canada; 2 University of British Columbia, Vancouver, Canada; 3 Karolinska Institutet, Stockholm, Sweden

**Keywords:** euthanasia, policy, medical assistance in dying, delivery of health care, ethics, policy, review, Canada

## Abstract

The purpose of this study was to describe policies developed by English-speaking Canadian health authorities to guide multi-disciplinary healthcare practice in the context of MAID. Seventeen policies from 9 provinces and 3 territories were identified and analyzed thematically. Themes developed from these documents related to ensuring a team approach to care, supporting informed patient choice, creating region-specific guidance on eligibility criteria and safeguards, accommodating conscientious objection, and making explicit organizational responsibilities. Ethical language concerned vulnerability, non-judgmental care, dignity, non-abandonment, confidentiality, moral conscience, and diverse cultural values. Overall, these policies addressed important risk mitigation strategies, acknowledged important social contracts, and supported ethical practice. Collectively, these policies outline important considerations in the evolving Canadian context for other jurisdictions seeking to create policy around assisted death.

## Introduction

The legalization of Medical Assistance in Dying (MAID) in Canada in 2016 has had important legal and ethical implications for healthcare institutions. Prior to 2016, any form of assisted suicide was strictly prohibited under Section 241 of Canada’s Criminal Code. In February 2015, the Supreme Court of Canada struck down the prohibition because it violated section 7 of the Canadian Charter of Rights and Freedoms, namely an individual’s right to life, liberty, and security. With this ruling, the Canadian government had 1 year to craft legislation to regulate MAID federally. The federal government could have opted out of writing this legislation, leaving the onus on the provinces and territories to implement and regulate MAID. Instead, the federal government chose to amend the criminal code under Bill C-14, thus creating common eligibility criteria and safeguards across the country. To date, MAID is the only healthcare service that is legislated at the federal level. In Bill C-14 (2016), MAID is defined as

*(a) the administration by a medical practitioner or nurse practitioner of a substance to a person, at their request, that causes their death; or (b) the prescribing or providing by a medical practitioner or nurse practitioner of a substance to a person, at their request, so that they may self-administer the substance and in doing so cause their own death (“Bill C-14: An Act to amend the Criminal Code and to make related amendments to other Acts (*medical assistance in dying),” 2016).

Under Bill C-14, only those whose natural death was reasonably foreseeable were eligible for MAID. This eligibility criterion was subsequently challenged under two sections of the Canadian Charter of Rights and Freedoms: section 7 (life, liberty, and security) and section 15 (equality), which specifies that every individual has the right to equal protection under the law without discrimination of mental and physical disability (Canadian Charter of Rights and Freedoms, 1982). In short, excluding those whose natural death was not reasonably foreseeable, challenged the liberty security, integrity, and autonomy of the person. After extensive public consultation, Bill C-7, which repeals the requirement that an individual’s natural death be reasonably foreseeable, was introduced and passed on March 17, 2021. This required the introduction of two sets of safeguards, one for persons whose natural death is reasonably foreseeable and one for persons whose natural deaths is not reasonably foreseeable (See Table 1 for eligibility criteria; Nicol & Tiedeman, 2021).

**Table 1.** Changes in Common Eligibility Criteria for MAID in Canada Bill C-14 and Bill C-7.

Eligibility under section 241.2(3) of the criminal code (C-14)	Updated eligibility under Bill C-7 (2021)
Be eligible for health services funded by a government in Canada (section 241.1(1)(a))	No change
They are at least 18 years of age and capable of making decisions with respect to their health (section 241.1(1)(b))	No change
The person has a grievous and irremediable medical condition that meets all of the following criteria;	Reasonably foreseeable natural death clause repealed
Serious and incurable disease or disability	
Advance state of irreversible decline	
Enduring physical or psychological suffering	
Natural death has become reasonably foreseeable (section 241.2(a))	
The person has made a voluntary request without external pressure 241.1(d)	No change

As Canadian health care is regulated and delivered at the provincial/territorial level, implementation of this legislation became the responsibility of the regional health authorities in each of Canada’s 13 provinces and territories. These regional health authorities are structured either as a single authority that governs an entire province/territory or through regional authorities responsible for specific geographical contexts within a province. Health authorities must ensure that their MAID services are consistent with the federal law; however, they decide how to deliver those services and have the authority to enact additional regulation. For example, some provinces have developed a central MAID coordinating service that relies on primary care providers while others have developed a MAID-specific team that provides MAID assessments and provisions.

Although there is no federal regulatory committee that oversees MAID, in 2018 Federal regulations came into effect requiring the monitoring of MAID through physician and nurse practitioner self-report (Government of Canada), guidelines which were updated most recently in 2022 (Government of Canada, 2022). Provinces have developed different ways to manage oversight. For example, British Columbia, Alberta, Saskatchewan, and Manitoba have established review committees to ensure MAID is provided in accordance to the provincial and federal regulations (Health Canada, 2020). Professional regulatory bodies and disciplinary colleges for physicians/surgeons, pharmacists, and nurses at various provincial, territorial, and federal levels also play an important role in ensuring that healthcare providers abide by safe, legal, and standard care practices (Health Canada, 2020).

An important way to gain insight into the development of MAID in Canada is through an exploration of policy documents created by health authorities to guide practice. These institutional policy documents perform a number of functions including mitigating risk, promoting standardization, and guiding best practices. These policies constitute a social contract between an institution and its stakeholders (Goodridge, 2010) that can be deeply ideological in that they reflect the cultural context within which they are created (Boychuk, 1999). Policies, especially in areas that are sensitive and value-laden, can be a mechanism of social change. One example of the way that policies influence ideas and practices were the baby-friendly hospital policies initiated in the early 1990s that limited that use of infant formula in hospital and hence, supported a social movement toward exclusive breastfeeding (Heinig, 2010).

Institutional policies are particularly important in the context of morally contentious practices such as patients refusing blood transfusions (Winkler, 2005), or end-of-life practices to relieve suffering such as palliative sedation and euthanasia. Formal institutional policies in these situations perform a number of functions: they reduce legal risk by clearly specifying a standard of practice (Cranford & Gensinger, 2002), they reduce the individual moral burden on practitioners by creating common ethical standards (Winkler, 2005), and perform an educational function in the case of moral uncertainty (e.g., differences between euthanasia and palliative sedation; Cranford & Gensinger, 2002). Despite the importance of these policies, a scoping review of barriers and challenges of implementing MAID indicated that institutional guidelines are often “underdeveloped or poorly integrated into existing health services” (Fujioka et al., 2018, p. 210). An earlier overview of MAID literature, policies, and reports revealed significant differences across Canada (Silvius et al., 2019).

Although there are few empirical studies of the impact of policies on practice, there is some indication that policies in morally contentious areas provide important support to healthcare providers (Lemiengre et al., 2007); early studies of nurses’ experiences with MAID in Canada indicated that a dearth of policies left nurses feeling unsupported in this new area of practice (Pesut, Thorne, Schiller, Greig, & Roussel, 2020). However, there is other evidence to suggest that such policies have little impact on day-to-day practice (Goodridge, 2010). Further, even policies that deal with morally contentious areas may lack explicit ethical underpinnings (Lemiengre et al., 2007). With this in mind, the purpose of this study was to explore policies developed by English-speaking, Canadian health authorities to guide multi-disciplinary healthcare practice in the context of MAID. It is important to note that in Canada that are also many faith-based organizations that provide healthcare services, particularly in the area of palliative care. These organizations are publicly-funded to varying degrees. This review does not consider policies from these organizations.

## Methods

This was qualitative content analysis of policy documents (Fisher & Hamer, 2020). The research questions were: (1) What content have health authorities determined to be important in providing direction to healthcare providers participating in MAID? (2) What are the variabilities across these documents? (3) What explicit ethical guidance is provided? Policies were collected between October 2020 and February 2021 just prior to the revision of MAID legislation under Bill C-7.

### Search Strategy

A three-step search strategy was used to identify approved institutional policies that provided direction for MAID practice from a possible sample of 22 English speaking, publicly-funded, and non-faith-based health authorities: (1) an initial internet search of health authority websites, (2) followed by an email request, and (3) a freedom of information request under the Federal Access to Information Act. The initial internet search yielded documents from thirteen health authorities (see Table 2 for a description of the documents). For those health authorities whose documents were not publicly available online, an email request was sent to the regional/provincial MAID contact. Two reminder emails were sent to regions that did not respond. This strategy yielded an additional four documents. As a last strategy, a freedom of information request was sent. This yielded no additional documents. These regions did not supply their documents with the explanation that they were either currently under revision or had been withdrawn from the public domain.

**Table 2.** List of Policy Documents Retrieved.

Province	Health authority	In-text	Year published	No. of pages
Alberta	Alberta Health Services	AHS	2018	12
British Columbia	British Columbia Ministry of Health	BCMOH	2018	6
	Interior Health Authority	IHA	n/a	9
	Island Health	IH	2020	5
	Northern Health Clinical Guidelines	NHa	2020	12
	Northern Health LPN/RN/RPNs Aiding a physician or nurse practitioner Clinical Practice Standard	NHb	2020	9
	Vancouver Coastal Health	VCH	2018	22
Manitoba^a^	Interlake-Eastern Regional Health Authority	IERH	2020	10
	Prairie Mountain Health	PMH	2019	7
	Southern Health/Santé Sud	SH	2019	9
	Winnipeg Regional Health Authority	WRH	2018	9
New Brunswick	Horizon Health Network	HHN	2017	9
Northwest territories	Government of Northwest Territories, Department of Health and Social Services	NWT	2018	19
Nova Scotia	Nova Scotia Health Authority	NSH	2019	23
Nunavut	Government of Nunavut Department of Health	NUN	n/a	10
Ontario^ab^	Center for Effective Practice (CEP)	ON	2019	11
Yukon	Yukon Health and Social Services	YT	2016	2

a Two health regions contained an extensive disclaimer on their documents indicating the they were a guide only and did not constitute medical or professional advice.

b At the time of writing Ontario’s health care system was undergoing restructuring and amalgamating from 20 health agencies to a single agency called Ontario Health. This Resource was developed as part of the Knowledge Translation in Primary Care Initiative and was funded by the Government of Ontario. The development of this Resource was led by the Center for Effective Practice (CEP) with collaboration from Ontario College of Family Physicians and Nurse Practitioners Association of Ontario.

Inclusion criteria were approved policy documents that provided direction for MAID practice from English speaking, publicly-funded health authorities. Exclusion criteria were policy documents from faith-based, French-speaking and Indigenous health authorities. Documents from Quebec, which is a French-speaking province, were excluded primarily because of the differences in the law directing MAID. Faith-based health region documents were excluded because many do not permit MAID in their institutions. Indigenous regional documents were excluded because there were no research agreements in place with Indigenous partners and a comparison with non-Indigenous regions would have been culturally inappropriate.

### Analysis

Analysis followed the principles of qualitative description (Sandelowski, 2010). NVIVO^QSR^, a qualitative software program, was used to aid analysis of the documents. Two investigative team members read through the documents in their entirety to construct open codes that reflected the content. The open codes were then used to code several documents and were further refined based upon this initial coding. These refined codes were then used to code the entire set of documents. Analysis meetings with the principal investigator and three research team members were held to ensure consistency, confirmability, and credibility of findings.

## Findings

A total of 17 MAID policy documents spanning 16 health authorities were retrieved (see Table 2 for a list of documents). Sixteen of these documents were generic to all healthcare providers within a health authority and one document was specific to Registered Nurses.

Table 3 provides an overview of the codes and sub-codes across documents, derived from analysis. The six thematic categories are: the team approach, supporting informed choice, eligibility and safeguards, accommodating conscientious objection, and organizational responsibilities. Table 4 provides an overview of the common ethical language that was used in the documents.

**Table 3.** Themes in Canadian Medical Assistance in Dying Policies.

Theme	Sub-theme
Team approach	Assessor and Provider eligibility, competencies, roles, and responsibilities
	Central Coordination Services
	Required Education, advanced procedural privileges, establishment, and maintenance of competency
	Interdisciplinary team approach
	Restrictions and roles RN practice
	Importance of an interdisciplinary team approach
	Requirements of clinical operational teams
	Required numbers of person in attendance
	Restrictions and roles NP practice
Supporting Informed choice	Patients are able to make an informed choice about proceeding with MAID
	Self-Administration Death including risks, educational requirements for patients and family, contingency plans, obligations of healthcare providers
	Timely, coordination, and reasonable access
	Locations of death
	Post-death care (estate planning and life insurance, organ and tissue donation)
Eligibility and safeguards	Ensuring and corroborating informed choice and motivation for request (e.g., suffering)
	Procedures to gain further assessments if deemed ineligible
	Virtual/telehealth assessments
	Communication aids and potential accommodation
	Who can and must discuss MAID and to what extent?
	Details of what constitutes informed consent, assessing capacity, and ways of documenting
Accommodating conscientious objection	Definition of conscientious objection
	Responsibilities of health care providers who take stances on conscientious objection
	*Institutional Obligations*
	Pre-existing contractual arrangements with institutions that take stance on conscientious objection
	Rights under pre-existing contracts (e.g., provision and assessment)
	Organizational responsibilities to clients
Organizational responsibilities	Organizational oversight
	Provincial review committee
	Responsibilities toward patient and families
	Logistics around MAID Procedure
	Directives for family members after death
	Guidance to patients and families
	Cultural and spiritual care
	Debriefing and wellness supports
	Develop plans for conscientious objection

**Table 4.** Prevalent Ethical Language in Policies.

Terms	Descriptions
Vulnerability	Protection of vulnerable patients, considering influences on a patient’s choice of MAID; protecting vulnerable patients from discrimination, coercion, exploitation, and undue influence.
Non-judgmental care	Compassionate care; care without bias or discrimination; not impeding or denying MAID; patients free from experiencing negative attitudes and responses that leave them feeling unwanted or shamed; providers may not be confusing, coercive, or provide incomplete information; freedom from stigma
Dignity	Patient autonomy, relational autonomy, and self-determination; right to end suffering with dignity and respect
Non-abandonment	Duty to provide care; timely care; continuous care
Confidentiality	Strict confidentiality of patient choice; respect privacy of MAID request; no public comment about MAID request; particular attention to privacy and confidentiality necessary for MAID
Moral conscience	Acting in good faith; respecting personal beliefs and values
Cultural values	Respecting diverse beliefs; respecting cultural, linguistic, and spiritual or religious ties and beliefs; culturally competent

### The Team Approach

A prominent theme in these policies was describing which healthcare professionals could be involved in MAID. Those eligible to act as assessors and providers were physicians and nurse practitioners, although in one region, nurse practitioners could not act as providers because they were not legally permitted to complete the required documentation of death (Winnipeg Regional Health Authority [WRH], 2018). Assessors are those individuals who can determine whether persons applying for MAID meet eligibility criteria; providers are those individuals who administer or prescribe the medication to cause death. In all health authorities in one province (Interlake-Eastern Regional Health Authority (IERH), 2020; Prairie Mountain Health [PMH], 2019; Southern Health [SH], 2019; WRH, 2018) only those with the title Authorized Practitioner and who were part of the provincial MAID Team were allowed to act as assessors and providers. In this province, MAID requests needed to be communicated to the MAID team, a resource available to both healthcare providers and patients. In some policies MAID assessors and providers were required to have advanced procedural privileges (Island Health [IH], 2020; Northern Health [NH], 2020a). Elsewhere, central coordination services figured prominently across the policies. The primary role of the central coordination service was to facilitate timely access to MAID by linking applicants to assessors and providers.

The role of the registered nurse was relatively invisible in these policies even though legally they are considered part of the MAID team if they perform specific roles. Policies indicated that nurses who are involved must possess the necessary clinical skills (e.g., intravenous initiation; Interior Health Authority [IHA], n.d.; NH, 2020b; Vancouver Coastal Health [VCH], 2018) and be able to identify holistic needs of the patient and family (IHA, n.d.). Notably in one region, registered nurses were required to achieve 80% on a competency exam specific to MAID to assist with MAID provision within their scope of practice (NH, 2020b). In many policies, registered nurses were able to engage in conversations about motivations or care concerns surrounding a MAID request and were required to notify a most responsible provider in the event of a patient request for information (VCH, 2018; Yukon Health and Social Services [YT], 2016). Other policies limited registered nurses’ role by prohibiting them from pronouncing death related to MAID, participating in any aspect of medication handling or administration, or restricting conversations with patients about MAID. (NH, 2020b; VCH, 2018)

The importance of an interdisciplinary team approach was specified in several documents. The team could include physicians, nurses, and pharmacists, unregulated care providers and allied health professionals (e.g., VCH, 2018). Other documents outline who can be part of the team: “The team is interdisciplinary and comprised of physicians, pharmacists, nurses, social workers, speech language pathologists and other allied health professionals” (WHR, 2018, p. 6). Important roles of these teams were to provide ongoing care of patients, consult on cases, discuss roles and responsibilities, and conduct case reviews and debriefing. Team members were encouraged to support one another in self-care in acknowledgement that the work itself can be “overwhelming and emotional” (Ontario Centre for Effective Practice [ON], 2019, p. 6; WRH, 2018, p. 6). Those involved were encouraged to seek out wellness supports. In consideration of the nature of this work, one document specified or recommended that two persons must attend a MAID death (NH, 2020a).

### Supporting Informed Choice

Ensuring that patients were enabled to make an informed choice about MAID was a central concern across policies. Documents outlined requirements for ensuring that patients understood their health status and prognosis, other options available to them, the MAID process, and the certainty of death as a result of the provision. Patients were also required to be informed of the two methods of MAID, provider administration or self-administration (although in one region, the self-administration medication was not approved; WRH, 2018). Some policies required providers to discuss factors associated with self-administration with the patient and their family (ON, 2019) and required them to be prepared for what to expect with a self-administered death; others required a practitioner be present during the self-administration process (Government of Nunavut [NUN], n.d.; Government of Northwest Territories [NWT], 2018; NH, 2020a; VCH, 2018); and one required a contingency plan should the self-administration procedure be unsuccessful (Alberta Health Services [AHS], 2018). This final requirement was particularly important when healthcare providers were not present at the death. In one health region, if explicitly requested by a patient, family members were permitted to assist or provide the lethal medication without facing criminal charges (NUN, n.d.). Another document emphasized that there was “no legal requirement to inform family of a patient’s decision to pursue MAID” (WRH, 2018, p. 5).

Policies further promoted patient choice in the timing and location of death. Statements such as “timely and reasonable” access to MAID (AHS, 2018, p. 2) reflected a commitment to patient choice. Overall, policies directed practitioners to accommodate a patient’s wishes; and, in some health authorities with large rural and remote areas, providers were encouraged to provide services as close to home as reasonably possible (NUN, n.d.; NSH, 2019). In all policies, patients had choice over who could attend their death, including family, friends, and caregivers. Choice also extended to post-death planning. Two health authorities encouraged patients to seek legal advice regarding estate planning and life insurance for end-of-life wishes (NUN, n.d.; NWT, 2018). Except for one policy (ON, 2019), documents did not discuss organ and tissue donation.

### Eligibility and Safeguards

All policies outlined federal eligibility and safeguards for MAID, including how to approach requests for MAID and how to corroborate informed consent. Policies further discussed a need for assessors to make a careful exploration of a MAID request and to actively listen to a patient’s concerns related to unmet care needs. Several policies explored assessing suffering as eligibility criteria for MAID (Horizon Health Network [HHN], 2017; IH, 2020; NH, 2020a; ON, 2019; VCH, 2018; WRH, 2018), though one policy emphasized that suffering could not be rooted in a mental health condition (VCH, 2018) and another highlighted the importance of acknowledging psychological suffering (HHN, 2017). Multiple policies described ways in which patients could seek additional assessments if the original assessment deemed them ineligible. In consideration of the rural and remote regions of Canada, several policies permitted eligibility assessments to be conducted virtually (NUN, n.d.; NH, 2020a, 2020b; Nova Scotia Health Authority [NSH], 2019; VCH, 2018).

A number of policies referenced communication aids to guide conversations about MAID with patients. These aids ranged from general statements to use during conversations to specific guidelines (NH, 2020b) or checklists to facilitate patient conversation (VCH, 2018; WRH, 2018). However, an important part of these policies was outlining who was permitted and/or obligated to communicate with patients about MAID. In most health authorities, if a patient inquired about MAID, any health care provider (e.g., social workers, psychologists, care aides, etc.) could respond by sharing general information or resources (e.g., websites, information packages, or contact cards) in relation to end-of-life care, including MAID, before referring them to a physician or nurse practitioner. However, in one instance trained physicians and nurse practitioners were allowed to initiate conversations about MAID with patients; indeed, it was described as a “professional obligation” (IHA, n.d.). Other policies, however, assumed that patients would initiate the conversation and cautioned healthcare providers not to incite or recommend MAID to patients (NUN, n.d.; NWT, 2018; HHN, 2017; IH, 2020; IHA, n.d.; NH, 2020b; NSH, 2019; ON, 2019; VCH, 2018; WRH, 2018).

Full and informed consent was discussed in all policies. Informed consent required that patients fully understand the finality, expectations, and potential complications surrounding a MAID death, and this was especially discussed in reference to a self-administered death. In some policies, providers were encouraged to consult with colleagues or specialists (i.e., psychiatrists) if patient capacity to provide informed consent was in doubt (NUN, n.d.; NH, 2020a; NWT, 2018; ON, 2019; WRH, 2018). Policies also provided specific direction to practitioners regarding ways of documenting informed consent, including the need to accommodate those who are unable to provide written consent (AHS, 2018; HHN, 2017; IHA, n.d.; NUN, n.d.; NH, 2020a; NWT, 2018; ON, 2019; VCH, 2018; WRH, 2018). Some policies also directed practitioners to assessment tools and resources to help determine consent and capacity (NSH, 2019; ON, 2019; WRH, 2018).

### Conscientious Objection

Every policy reviewed discussed the issue of personal and institutional conscientious objection to some degree. Conscientious objection was defined as a provider who was unwilling or unable to provide MAID due to reasons of thoughts, conscience, or religion. Conscientious objection was not necessarily limited by religious beliefs but rather anchored in moral and ethical beliefs that in some cases were defined as being deeply held. A number of responsibilities were outlined for those healthcare providers who took the stance of conscientious objection. They were required to make their stance known to their manager in a timely manner to minimize disruptions or burdens upon patients and other providers. Other responsibilities included how much information conscientiously objecting healthcare providers were required to provide to a patient. This ranged from providing publicly available information such as the coordination service contact (e.g., NWT, 2018) to ensuring an effective referral to a healthcare provider willing to conduct an assessment for MAID (e.g., HHN, 2017; ON, 2019). The most important responsibilities were non-abandonment and duty to provide ongoing care. Only in one policy, did conscientiously objecting persons have a choice to no longer provide continuous care (AHS, 2018). For example, a health care provider could be “unwilling or unable to support the provision of either the patient’s usual care or care specific to Medical Assistance in Dying” (AHS, 2018, p. 8).

The policies reviewed also addressed the rights and responsibilities of institutions to conscientiously object to assessing and/or providing MAID within their institutions. These were typically faith-based facilities with contractual agreements with health authorities to provide care. In several policies, long-standing denominational health agreements were cited as providing guidance for the subsequent policy (HHN, 2017; IH, 2020; IHA, n.d.; NH, 2020a; VCH, 2018). Conscientiously objecting organizations had the choice of the degree to which they would participate in the MAID process (British Columbia Ministry of Health [BCMOH], 2018; IH, 2020; VCH, 2018); but some specified that organizations were required to provide eligibility assessments for MAID onsite (IHA, n.d.) while others were not (IH, 2020). Providing continuing care to clients who request MAID was emphasized (e.g., IERH, 2020; SH, 2019). In this case, external providers would perform MAID. A responsibility of these organizations was to post their policies publicly so that patients could make an informed choice about the options available to them should they choose to enter the facility (IERH, 2020; PMH, 2019; SH, 2019). Healthcare providers who worked in these facilities had to abide by the same responsibilities described under conscientiously objecting individuals.

### Organizational Responsibilities

Organizational responsibilities were described across policies in relation to oversight, consistency of care for patients and family, and healthcare provider well-being. To ensure oversight of the MAID process, provincial and territory-level review committees were referred to in policies (AHS, 2018; NUN, n.d.; NWT, 2018; IERH, 2020; OMH, 2019; SH, 2019). These committees are responsible for reviewing, auditing and investigating MAID cases and ensuring that reporting requirements were fulfilled under federal and provincial/territorial legislation. In one location, a review committee was activated every time there was a request for a self-administered death (NUN, n.d.). To inform quality and system improvements, two regions reviewed MAID cases and provider/assessor documentation (NUN, n.d.; NH, 2020a).

Part of this oversight included ensuring a common standard of care across regions. For example, in BCMOH (2018) directed an overarching policy to the five regional health authorities to ensure consistency in care across the province. Although individual health authorities have autonomy in shaping their operational policies, local policies must at minimum reflect service delivery outlined in the provincial policy.

Several policies outlined organizational responsibilities toward patient and family. One health region explicitly stated that organizations should ensure that patients and family were prepared for what to expect once the patient had received MAID (ON, 2019). Policies ranged from having a thorough discussion about the date, time, and location of the provision with the patient (WRH, 2018) to specific instructions for family/caregivers on what to do once the patient had died (e.g., call the coroner’s office; ON, 2019). Honoring and providing appropriate cultural and/or spiritual care was mentioned in five policies (AHS, 2018; NSH, 2019; NUN, n.d.; NWT, 2018; VCH, 2018) and among these policies, directives ranged from briefly mentioning that patients should be ensured access to cultural/spiritual care (AHS, 2018; NWT, 2018; VCH, 2018), to outlining that MAID practitioners must act in accordance with “Inuit Qaujimajatuqangit (IQ/Inuit) societal values (ISV)” (p.8) at all times throughout the MAID process and emphasizing the importance of respecting the cultural, linguistic, and religious/spiritual considerations of the person receiving MAID (NUN, n.d.).

The wellbeing of health care providers was also recognized as important and most policies included a discussion of the role of leadership (directors/managers of units, facilities etc.) in supporting staff/practitioner wellbeing. Policies outlined the responsibilities of leaders in ensuring that staff had the opportunity to debrief after a MAID death. Some policies simply referred to practitioners and care teams having the opportunity to debrief (IHA, n.d.; NSH, 2019; ON, 2019; VCH, 2018; WRH, 2018). Other policies provided more extensive direction about the psychological impact of MAID on staff (ON, 2019; WRH, 2018). In two policies, MAID was described as an overwhelming or emotional experience for some staff (ON, 2019; WRH, 2018). Another policy suggested staff could access formal psychological support through Employee Health Services or other mental health programs (HHN, 2017). Likewise, some policies (IH, 2020; IHA, n.d.; VCH, 2018) directed those in leadership positions to develop procedures to support patient requests for MAID while accommodating staff who were conscientious objectors. Some policies (IH, 2020; IHA, n.d.; NHa, 2020) emphasized procedures for accommodating conscientiously objecting staff (e.g., keeping records and developing standards) while others emphasized ensuring quality care for patients (e.g., access and duty to care; NH, 2020b; VCH, 2018; YT, 2016).

### Ethical Concepts

Five ethical concepts were used consistently across documents: vulnerability, non-judgmental care, dignity, non-abandonment, and confidentiality. Two additional concepts focused on the moral conscience of healthcare providers and the need for recognizing diversity (See Table 4). Further, there was language directing healthcare providers to consider factors that might unduly influence patients’ choice of MAID, an important factor in the legislation (e.g., AHS, 2018; IH, 2020; NSH, 2019; ON, 2019; VCH, 2018; WRH, 2018; YT, 2016). The concept of non-judgmental care reflected attention to the potential stigma that patients choosing MAID might experience in healthcare environments (NSH, 2019). Healthcare providers were cautioned to provide care without bias or discrimination (e.g., AHS, 2018; NSH, 2019; VCH, 2018). The term dignity was commonly used in the documents alongside language of autonomy and rights (e.g., AHS, 2018; HHN, 2017; NUN, n.d.; NWT, 2018; NH, 2020b; NSH, 2019). This reflected the common understanding that MAID is meant to provide death with dignity. Finally, the issue of confidentiality was addressed in a number of documents (e.g., NUN, n.d.; IH, 2020; IHA, n.d.; NH, 2020b; NSH, 2019; VCH, 2018), directing healthcare providers to respect the absolute privacy of individual patients. While most ethical language was directed toward the care of patients, documents acknowledged the importance of the moral conscience (e.g., AHS, 2018; NH, 2020b; NSH, 2019; VCH, 2018) of healthcare providers, above and beyond their right to conscientiously object to participating in MAID. This language explicitly recognized the importance of healthcare providers’ personal beliefs and values in the context of practice and their freedom to act in good faith in relation to those beliefs. While the majority of these uses of ethical language reflected a common approach across persons (e.g., dignity and vulnerability), some documents emphasized the need to consider cultural (e.g., AHS, 2018; NUN, n.d.; NWT, 2018; VCH, 2018), linguistic, and spiritual diversity and the importance of healthcare providers being competent in a culturally safe approach. This theme recognized that important ideas such as dignity and vulnerability might be expressed in a variety of manners within different contexts.

## Discussion

The purpose of this study was to describe policies developed by Canadian health authorities to guide multidisciplinary healthcare practice in the context of MAID. The subsequent discussion will focus on the key functions of health policy: mitigating risk, acknowledging social contracts, and supporting ethical practice with an emphasis on the implications for nursing practice.

### Mitigating Risk

Mitigating risk is particularly important in the context of MAID which is the only healthcare treatment in Canada governed by federal law. Policies reviewed in this study addressed several areas relevant to mitigating risk: healthcare provider competency development, patient choice without coercion, and additional safeguards around self-administration.

Developing competency in the area of MAID has been an ongoing challenge in Canada. An important decision for each province and territory has been how to set up MAID services in a way that balances safety with accessibility (Wiebe et al., 2021). One of the initial challenges was accessibility to MAID (Oczkowski et al., 2021a, 2021b), in part, because of the difficulties in finding sufficient MAID assessors and providers across the vast geographic distances of Canada (Pesut, Thorne, Wright, et al., 2021; Wiebe et al., 2021). Many primary care providers chose not to act as MAID assessors and/or providers for a variety of reasons in addition to conscientious objection. Barriers to participation included adequate time and/or remuneration (Oczkowski et al., 2021a, 2021b; Pesut, Thorne, Schiller, Greig, & Roussel, 2020) and other factors such as emotional labor, comfort with death, and personal experiences (Brown et al., 2021). One way to ensure accessibility and a common approach to care was to establish MAID teams responsible for coordinating and/or assessing and providing MAID within a specific geographic region—in some cases spanning entire provinces. This ensured a body of professionals who became competent in this care, an important consideration when studies indicated that healthcare providers reported poor knowledge of MAID policies and practice (Ball et al., 2019; Oczkowski et al., 2021a, 2021b). However, the lack of specific guidance regarding competency for registered nurses who participated in MAID was lacking in these documents. This is an important oversight considering that nursing regulatory documents set out specific requirements for nurses’ knowledge and competency in the context of MAID (Pesut et al., 2019).

One of the most challenging competencies has been determining whether a client is eligible for MAID. Legislated eligibility criteria in Canada have been criticized for being unclear, (Downie & Scallion, 2018) and thus requiring clinical interpretation, (Canadian Association of MAiD Assessors and Providers, 2017) meaning that assessors must exercise an undue degree of subjective judgment when deeming what they perceive to be an ethical interpretation of the law (Gupta & Blouin, 2022; Pesut, Thorne, Wright, et al., 2021; Pesut, Wright, Thorne, et al., 2021). Evidence has suggested that there has been variability in MAID assessors’ determination of eligibility, that clinicians draw upon a variety of sources in making that determination, and that the understanding of the criteria has broadened over time (McMorrow et al., 2020). Hence, the establishment of MAID teams was one way to mitigate risk. Policies described in this review used a number of other strategies to mitigate risk such as establishing educational requirements for primary care providers and nurses involved in MAID; requiring reporting to MAID coordination services; and designating who can be involved in MAID, even to the point of requiring procedural privileges which had to be granted by the health region. Although registered nurses are not responsible for determining eligibility for MAID, there are nursing regulatory documents that indicate that nurses should be satisfied that the eligibility criteria and safeguards have been met prior to their participation and so these same challenges would affect their practice (Pesut et al., 2019).

Another important risk mitigation factor addressed in these documents was ensuring that patients had free, informed, and uncoerced choice regarding MAID. This is a balance between ensuring that patients know about MAID and how to request it while ensuring that those choices are free from coercion (Duong, 2021). Policies described in this review took various approaches to mitigating this risk including providing direction for exploration of the MAID request, developing communication aids, and outlining the types of suffering that would deem a person eligible. Policies also provided direction about what needed to be included in those conversations (e.g., health status and other options for care). An interesting finding was who was allowed to engage in these conversations. While it was generally accepted practice that any healthcare provider could respond by sharing information or resources, in some cases, only those healthcare providers trained in these conversations could take part, and several policies cautioned about not inciting or recommending MAID to patients. Evidence to-date indicates that although many nurse practitioners have developed nuanced communication strategies in the context of MAID, registered nurses are confused about what they are allowed to say and when (Pesut, Thorne, Schiller, Greig, Roussel, & Tishelman, 2020). An ongoing debate in the Canadian landscape is whether MAID should be introduced to all persons by healthcare providers as part of advance care planning (Seller et al., 2019). Whereas initially when the legislation was introduced this might have been perceived as recommending MAID, more practitioners are assuming that providing MAID education as part of advance care planning is part of ensuring informed choice for patients. (Canadian Association of MAiD Assessors and Providers, n.d.; Pesut, Thorne, Wright, et al., 2021)

The final notable risk mitigation evident in these policies was around self-administration of the medication that leads to death. For example, nursing regulatory documents delineate clear rules around what registered nurses can do in the context of self-administration (e.g., hand the pills to the patient) so that they do not cross into the boundary of administering the medication (Pesut et al., 2019). Unlike practice in the United States where only self-administration is allowed, self-administration in Canada is a rare occurrence. Of the 7,595 reported MAID cases in Canada in 2020, only seven cases were self-administered (Health Canada, 2021). Initially, this was because practitioners were having difficulty getting access to the appropriate medications in Canada. However, providers have also been concerned about patients vomiting the medication or medical conditions that make absorption unreliable, both of which can lead to a difficult dying process (Ball et al., 2019). Therefore, risk mitigation contained in these policies included having a contingency plan if the self-administration was unsuccessful; this typically meant that healthcare providers needed to be present during self-administration. Other mitigation factors included patient and family education about what to expect and a discussion of risks so that they were fully informed. In at least one jurisdiction, self-administration was perceived to be so risky that a practice review was held in each instance.

### Acknowledging Social Contracts

Institutional policies act as social contracts between stakeholders. What was most notable in these policies were the documented responsibilities between an organization and its employees, the responsibilities between health regions and their faith-based healthcare provider partners, and between health regions and patient and family partners. A number of policies in this review addressed the responsibilities of health regions to support the well-being of healthcare providers involved in MAID. These included providing opportunities for debriefing after a MAID death, ensuring access to formal psychological support, and requiring that at least two persons are present for any MAID death. Such policies reflect the emotionally demanding work of providing care to those being assessed for, or receiving, MAID (Pesut, Thorne, Schiller, Greig, Roussel & Tishelman, 2020). Evidence around the delivery of MAID in Canada has recognized the importance of collegial support in doing this demanding work (Elmore et al., 2018; Pesut, Thorne, Storch, et al., 2020), and policies such as those described here recognize that need.

Additional employer responsibilities addressed protecting the rights of health care providers to conscientiously object to participation in MAID. This right is addressed in the Canadian legislation, but it is ultimately up to employers to place boundaries around that right. The right of a healthcare provider to conscientious objection must be balanced by the duty to the “life, liberty and security” of the patient (Christie et al., 2016, p. 9). Questions arising from this right include the degree to which healthcare providers are obligated to perform related care (particularly in the case of registered nurses as institutional employees) and to provide an effective referral to someone who will provide MAID. There is some evidence in the Canadian context that, taking the stance of conscientious objection is a difficult one for organizationally-employed registered nurses who may feel conflicted in their loyalties to patients and misunderstood by colleagues who are supportive of MAID (Lamb et al., 2019). So, while these policies acknowledged the right to conscientiously object, there was little guidance provided on how to negotiate these difficult situations, and in most cases, Registered Nurses were required to continue to perform all care except administering the MAID medication itself.

The challenges of individual conscientious objection are magnified in the context of conscientiously objecting institutions. In Canada, faith-based organizations are an important part of overall systems of care, particularly within palliative care. Once MAID was legalized, these organizations had to determine the degree to which they would participate in the MAID process. Would they allow assessments and/or provisions on site or would they require that patients requesting MAID be transferred to other institutions? Faith-based organizations took different approaches based upon the decisions of their denominational leadership. There is still debate within Canada about whether an institution should be afforded the same legal right to conscientiously object as an individual (Carpenter & Vivas, 2020; Downie, 2017). However, faith-based organizations have pre-existing social contracts with provinces and territories that allow them to carry out their faith-based traditions (De Bono, 2017), and the choices provided to faith-based organizations often reflect these long-standing agreements. If faith-based organizations chose not to offer MAID on site, a common approach in these policy documents was to require them to post their policies so that prospective patients would be aware of the limits of their individual choices at end-of-life. However, this approach does not solve the challenge for patients for whom the only available palliative care beds in their geographic area are located within faith-based facilities. Negotiating these difficult social contracts remains a work in progress.

Patients and family are the most important stakeholders in the MAID process, and a robust body of literature is developing around their needs—needs which are overwhelmingly related to care coordination and a patient-centered approach to care (Oczkowski et al., 2021a, 2021b). Patients and family want ready access to information about MAID, transparency around the process, anticipatory guidance, choice about location and timing, and emotional and tangible support for what can be a complicated and intense experience (Brown et al., 2020; Smolej et al., 2023; Thangarasa et al., 2022). Policies reviewed in this manuscript provide important guidance for healthcare providers in supporting informed patient choice, accommodating wishes regarding timing and location of death, obtaining full and informed consent, and providing anticipatory guidance. As nurses are often key members of the team in meeting these patient and family needs, a stronger emphasis on the nursing role in the policies is important.

### Supporting Ethical Practice

Policies have an important role in ethically contentious practices by creating an approach that serves to alleviate some degree of moral uncertainty (Lemiengre et al., 2010). However, as indicated in the introduction of this paper, there has been critique of policies for lacking explicit ethical underpinnings. Such was the case with the policies reviewed in this paper, although ethical language was prominent throughout these documents. Much of the language related specifically to eligibility and safeguard requirements. However, other language alluded to challenges that were likely to occur within the practice context with this morally contentious practice. For example, terms such as non-judgmental care, non-abandonment, and moral conscience suggested that deeply held beliefs about this practice could potentially lead to unethical care. Such terminological choices acknowledged that within the morally contentious landscape of MAID, it was important to reiterate the stances that should characterize relationships among professionals, patients, and family.

In the early days of MAID implementation in Canada there were significant concerns about the impact of healthcare providers’ values and beliefs in the clinical context. Those in support of MAID were concerned that conscientious objectors were acting as gate keepers to MAID access (Ball et al., 2019). Family of patients receiving MAID also experienced moral tension and judgment by clinicians at the bedside (Hales et al., 2019). As such, these policies were designed to provide a common reference point for morally acceptable behavior amidst the inevitable diversity of beliefs that characterize modern healthcare contexts. They set the standard for the tolerance and the “epistemic moral humility” that should characterize liberal democratic societies (Sulmasy, 2019, p. 7).

In this paper we chose to create a thematic account of ethical language, but one might argue that everything contained in these documents reflects a particular ethical perspective. Issues of patient choice, healthcare provider wellness, and structures that promote quality care are all ethically relevant considerations. As such, there may indeed be an important role for providing explicit ethical underpinnings within policy documents such as these. These explicit underpinnings are particularly important in light of the 2021 changes to the legislation that now extends MAID to those whose natural death is not reasonably foreseeable (Nicol & Tiedeman, 2021). Evidence to-date indicates that patients who do not have a reasonably foreseeable natural death may be choosing MAID, in part, because of vulnerabilities created through structural inequities and lack of support, creating difficult moral tensions in nurses and physicians (Marmoreo & Schneller, 2022; Pesut, Thorne, Wright, et al., 2021). An explicit ethical approach would provide importance guidance for nurses in these situations.

The study has several limitations. First, we were unable to collect a comprehensive set of documents. Second, several of these documents had disclaimers regarding practice guidance suggesting that these policy documents may not perform the same safety and contract functions that have characterized health system policy documents to date. This in itself was an interesting finding. Third, Bill C-7 came into effect after the collection of these documents and so policies would not have reflected the most recent legislation.

## Conclusion

This review of Canadian policy documents developed to guide interdisciplinary MAID practice can support policy makers in developing similar guidance documents in other countries in the process of implementing assisted death. Despite the variability of substantive content across these policy documents, taken collectively, they provide important domains of concern that can be applied to the contexts and cultures associated with various regional-specific policies. The following questions can inform this work: Who will constitute the team responsible for assisted death and how should they be prepared, regulated, and supported? How can the rights and responsibilities of conscientiously objecting persons and organizations be balanced to ensure high quality and accessible care for patients considering MAID? What additional regulations or processes need to be put in place to ensure patient choice and access? What organizational responsibilities are necessary to support high quality patient care and healthcare provider well-being? In the policies reviewed here, ethical language was embedded within the responsibilities outlined. However, organizations developing such policies might want to consider whether an explicit ethical framework might be useful in providing the foundation for such policies. Finally, it is important to note that some of the documents reviewed here took what we deem to be a cautious approach to the role that these policies should take in guiding clinical practice. Reflectively considering and making explicit the intended role of such documents may be an especially important consideration in ethically contentious and legally-regulated practices such as MAID.
